# Comparison of pregnancy outcomes and safety between cetrorelix and ganirelix in IVF/ICSI antagonist protocols: a retrospective cohort study

**DOI:** 10.3389/frph.2025.1492441

**Published:** 2025-04-14

**Authors:** Xinyue Peng, Xingyu Lv, Penghao Li, Yingxing Li, Yuling Hu, Yi Zhang, Yuan Li

**Affiliations:** ^1^ Department of Reproductive Medicine, Sichuan Jinxin Xinan Women and Children's Hospital, Chengdu, Sichuan, China; ^2^ Department of Scientific Research and Education, Chengdu Jinxin Research Institute for Reproductive Medicine and Genetics, Chengdu, Sichuan, China

**Keywords:** gonadotropin-releasing hormone antagonist, cetrorelix, ganirelix, controlled ovarian stimulation, live birth rate

## Abstract

**Introduction:**

This study aimed to compare the safety, efficacy, and clinical predictors of live birth outcomes between cetrorelix and ganirelix in GnRH antagonist protocols during controlled ovarian stimulation.

**Methods:**

In this retrospective cohort study conducted at a reproductive medicine center (June 2019–June 2022), 2,365 patients receiving cetrorelix (Group A) and 7,059 patients receiving ganirelix (Group B) were analyzed after 1:3 propensity score matching. Outcomes included LH surge suppression, OHSS incidence, endometrial morphology, embryological parameters, and live birth rates. Multivariate logistic regression identified independent predictors of clinical success.

**Results:**

Cetrorelix demonstrated superior LH surge control, with lower incidences of LH ≥10 U/L (4.9% vs. 7.6%, *p* < 0.001) and LH ratio(trigger day LH Gn day LH) ≥2 (6.1% vs. 9.2%, *p* < 0.001). Endometrial receptivity was more favorable in Group A, with higher Type A (66.2% vs. 60.1%) and lower Type C morphology (5.3% vs. 6.3%, *p* < 0.001). Ganirelix showed a higher overall OHSS incidence (1.1% vs. 0.4%, *p* = 0.01). Live birth rates were comparable (47.2% vs. 49.4%, *p* = 0.074). Multivariate analysis revealed advanced female age (≥35 years) reduced success (aOR = 0.65, 95% CI 0.57–0.74, *p* < 0.001), while AMH ≥4 μg/L (aOR = 1.29, 95% CI 1.02–1.64, *p* = 0.034), and dual embryo transfer (aOR = 1.51, 95% CI 1.38–1.65, *p* < 0.001) improved outcomes.

**Conclusion:**

Cetrorelix and ganirelix demonstrate comparable live birth rates and embryo quality, yet exhibit distinct safety profiles. Cetrorelix provides superior LH surge suppression and reduced OHSS risk, making it preferable for high-risk patients, while ganirelix may serve cases requiring rapid LH control. Cetrorelix offering enhanced endometrial receptivity (66.2% Type A morphology) and safety advantages. These findings support cetrorelix's role in optimizing ART safety without compromising efficacy.

## 1 Introduction

*In vitro* fertilization (IVF), embryo transfer, and intracytoplasmic sperm injection (ICSI) have allowed millions of couples facing infertility to achieve pregnancy. Recently, the number of cycles involving assisted reproductive technology (ART) has seen an upward trend in many areas (1). As ART continues to evolve rapidly, doctors worldwide are working to discover ovarian stimulation protocols that are both effective and safe. Controlled ovarian stimulation (COS) is a vital component of IVF/ICSI, enabling the collection of a large number of oocytes within a single cycle. The gonadotropin-releasing hormone antagonist (GnRH-ant) protocol, along with the standard long gonadotropin-releasing hormone agonist protocol, are the two most frequently employed stimulation protocols (1).

The World Health Organization (WHO) officially declared COVID-19 a global pandemic on March 11, 2020 (2), prompting countries to progressively impose lockdowns. China concluded its 3-year lockdown in December 2022. The pandemic caused major disruptions, presenting reproductive specialists with numerous challenges in patient care during this time. Consequently, many reproductive doctors in China began favoring the antagonist protocol for ovarian stimulation. This protocol has also gained international recognition due to its streamlined treatment process, reduced duration, fewer injections (3), and lower risk of ovarian hyperstimulation syndrome (OHSS) (4).

Moreover, the antagonist protocol offers benefits over the agonist protocol, including a shorter stimulation phase and reduced clinic visits, easing patient burden during the pandemic. With this transition, we have also examined methods to achieve clinical pregnancy and live birth rates using the antagonist protocol that are comparable to those of the agonist protocol. Furthermore, we seek to determine whether there are any variations in the safety and effectiveness between different antagonists.

Significantly, the European Society of Human Reproduction and Embryology (ESHRE) released guidelines for controlled ovarian hyperstimulation (COH) in 2020, stating that the GnRH-ant protocol can serve as a first-line treatment for patients with high, normal, or poor ovarian response. With COH safety in mind, the society's recommendations strongly advocate the GnRH-ant protocol as the preferred treatment for patients exhibiting a normal ovarian response (5). Moreover, the GnRH-ant protocol has seen increasing clinical use to prevent premature LH surges during COS prior to *in vitro* fertilization and embryo transfer (6).

GnRH antagonists, first discovered in the 1990s, work by competitively blocking GnRH receptors, leading to the rapid suppression of gonadotropin secretion (7). Additionally, ovarian hyperstimulation syndrome (OHSS) is an uncommon yet potentially life-threatening complication of COS (8). GnRH-ant directly inhibits gonadotropin release and prevents LH surges, reducing the incidence of OHSS by 10.0% compared to gonadotropin-releasing hormone agonist (GnRH-a) protocols (8).

The main antagonists currently used in China are Cetrorelix and Ganirelix. Cetrorelix was developed by Merck Serono and launched in 1999. It is now available in 45 countries, including those in Europe, entering the Chinese market in 2010. Furthermore, Hybio Pharmaceutical announced that it had received the “Drug Registration Certificate” for Cetrorelix Acetate Injection from the National Medical Products Administration (NMPA) on December 10, 2018. The availability of domestically produced Cetrorelix Acetate has provided clinicians and patients with more options.

Cetrorelix Acetate for Injection, a third-generation gonadotropin-releasing hormone antagonist, is widely used in the antagonist protocol and has been adopted by numerous large reproductive centers nationwide. However, there is a lack of clinical data regarding the efficacy and safety of Ferpront® (Ferring Pharmaceuticals, China), a domestic version of Cetrorelix. To meet the demand for evidence-based data among clinicians, our study aimed to compare the therapeutic effects of Cetrorelix (Ferpront®) and Ganirelix in IVF/ICSI, providing robust evidence for clinical use.

## 2 Materials and methods

### 2.1 Study design

This study was retrospective in design and included infertile patients who underwent *in vitro* fertilization embryo transfer or intracytoplasmic sperm injection (ICSI) at the Reproductive Medicine Department of Sichuan Jinxin Xinan Women and Children Hospital between June 2019 and June 2022.

#### 2.1.1 The primary inclusion criteria

1. Patients treated with IVF/ICSI fertilization
2. The female participants aged 20–45 years
3. Both ovaries present
4. Patients who obtain usable embryos using antagonist regimens
5. Patients using GnRH antagonist regimens between June 2019 and June 2022

#### 2.1.2 The primary exclusion criteria

1. Diagnosis of other system-related conditions, including thyroid disorders, diabetes, adrenal diseases, uncontrolled chronic illnesses, and conditions that are unsuitable for pregnancy.
2. Patients receiving ovarian stimulation for fertility preservation
3. Patients with premature ovarian failure
4. Patients undergoing genetic testing before embryo implantation
5. Patients who underwent Microsurgical Testicular Sperm Extraction.
6. Recent pre-treatment with other medications, such as oral contraceptives
7. Presence of sexually transmitted diseases

### 2.2 Procedures

The hospital ethics committee approved the retrieval of patient information. Baseline characteristics of participants were obtained from the hospital's information system, including age, body mass index, primary or secondary infertility, duration of infertility, cause of infertility, fertility treatment history, medical history, drug allergies, vital signs, physical examination, urine human chorionic gonadotropin (hCG) test, electrocardiogram, basal levels of follicle-stimulating hormone (FSH), luteinizing hormone (LH), estradiol (E2), prolactin, testosterone, progesterone (P), anti-Müllerian hormone (AMH), antral follicle count (AFC), complete blood count, urinalysis, blood biochemistry, coagulation profile, blood type, and screening for hepatitis B, syphilis, and human immunodeficiency virus (HIV), among others. Participants were divided into two groups based on the antagonist used during ovulation induction: Group A received Cetrorelix (Ferpront®, Ferring Pharmaceuticals, China), while Group B used Ganirelix (Orgalutran®, Organon, USA).

Controlled Ovarian Hyperstimulation (COH) was conducted using an antagonist approach. Treatment commenced on the 2nd or 3rd day of the menstrual cycle. Antagonists were introduced once the dominant follicle attained a size of 14–15 mm, or if the follicle's diameter exceeded 12 mm with serum estradiol levels above 300 pg/ml. Daily subcutaneous injections of 0.25 mg Cetrorelix or Ganirelix were given until the day of ovulation trigger.

Following this, COH was conducted using a flexible GnRH-ant protocol. Patients began COH on the 2nd or 3rd day of their menstrual cycle. Antagonists were administered when the dominant follicles reached 14 or 15 mm in diameter, or if their diameter was over 12 mm with serum estradiol levels exceeding 300 pg/ml. Daily subcutaneous injections of approximately 0.25 mg of either cetrorelix acetate (Cetrotide®, Merck Serono, Germany) or ganirelix acetate (Orgalutran®, Organon, USA/QingLe®, CHIA TAI TIANQING, Lianyungang, China) were given until the trigger day.

For patients at low risk of OHSS, 0.2 mg GnRH-a (Decapeptyl®, Ferring, Switzerland) along with 6,000 IU of HCG (Livzon Pharm, China) was used for triggering ovulation. In cases where patients were at high risk of OHSS (with E2 levels ≥5,000 pg/ml or ≥18 follicles greater than 14 mm in diameter), either 0.2 mg GnRH-a (Decapeptyl®, Ferring, Switzerland) or a combination of 2,000 IU of HCG (Livzon Pharm, China) and 0.2 mg GnRH-a (Decapeptyl®, Ferring, Switzerland) was administered.

### 2.3 Oocyte pick-up and embryo transfer

Oocytes were picked up 36–38 h after the trigger, and follicles with a diameter of ≥14 mm were extracted during egg retrieval surgery. Furthermore, embryo development was assessed on day three post-fertilization. A high-quality embryo is defined as having eight cells on day three post-fertilization, with blastomere fragmentation ≤20% and symmetry. Embryos are graded based on the level of fragmentation as follows: Grade I: <5%, Grade II: 5%–20%, Grade III: 20%–50%, and Grade IV: >50%. On day 3, embryos with 7–9 cells, <20% fragmentation, and uniform cell size were considered good in quality. The quality of the blastocyst was evaluated following the Gardner grading system, assessing the trophectoderm and the inner cell mass quality. For patients meeting the criteria for fresh embryo transfer, a cleavage-stage embryo was transferred on day three post-retrieval or a blastocyst was transferred on Day five post-retrieval. The remaining embryos were cultured to the blastocyst stage and then cryopreserved.

Patients who met the criteria for fresh embryo transfer, starting from the second day after oocyte pickup, received vaginal progesterone sustained-release gel (90 mg daily, Merck Serono, Germany) or progesterone capsules (Utrogestan®, 200 mg three times daily, Besins, Belgium), in addition to oral dydrogesterone tablets (Duphaston®, 10 mg three times daily, Abbott, USA) for luteal support. All patients underwent embryo transfer (ET) under ultrasound guidance. Approximately 14 days after ET, a beta-Hcg (bhCG) test was conducted. Patients who tested positive for pregnancy continued with luteal support. Two weeks later, for those with at least one intrauterine gestational sac with a detectable fetal heartbeat, the dosage of luteal support medications was gradually reduced. These support medications were discontinued at 10 weeks of pregnancy.

Furthermore, when the number of oocytes retrieved was >20, serum estradiol levels on the trigger day exceeded 3,500 ng/ml, progesterone during stimulation was greater than 1 ng/ml, or the endometrium was classified as grade C during stimulation, fresh embryo transfer was canceled. Patients who did not meet the criteria for fresh embryo transfer underwent frozen embryo transfer. Frozen embryo transfer, a hormone replacement cycle started from day 2–5 of menstruation. The patient received Estradiol Valerate, Progynova® (Bayer, Germany), 3 mg orally twice daily for 12 days, followed by ultrasound monitoring. When the endometrial thickness reached 8–14 mm or the optimal thickness for that patient, luteal support was initiated using either 90 mg of progesterone sustained-release gel, (Crinone®, Merck, Germany), once daily administered through the vaginal or 200 mg of progesterone capsules, (Utrogestan®, Besins Healthcare, Monaco), three times daily through the same route, in addition to 10 mg of dydrogesterone tablets, (Duphaston®, Abbott Laboratories, USA), taken orally three times daily to induce endometrial transformation. Cleavage-stage embryos were transferred on the third day of endometrial transformation, and blastocysts were transferred on the fifth day.

### 2.4 Follow-up plan

Patients were followed up on the registration day, trigger day, ET day, 7 days after oocyte pickup, 15 days after ET, 28 days after ET, and at 12 weeks of pregnancy.

### 2.5 Outcomes

#### 2.5.1 Primary outcomes

The primary outcome was the live birth rate (LBR). “Whether live birth occurred” was the outcome indicator of the multivariate logistic regression.

#### 2.5.2 Secondary outcomes

The secondary outcomes were LH suppression, OHSS incidence and pregnancy rates.

#### 2.5.3 Safety outcomes

The safety outcomes were Adverse events, OHSS.

### 2.6 Statistical analysis

Statistical analyses were performed using SPSS version 26.0 and R version 4.2.1.

#### 2.6.1 Descriptive analysis methods

Count data were expressed as frequency (*n*) and percentage (%), and the Chi-square test or Fisher's exact test was used for inter-group comparisons. Furthermore, measurement data that were normally distributed were described using mean and standard deviation (Mean, Standard Deviation). However, for those that were not normally distributed, the median (Median), the 25th percentile (Q1), and the 75th percentile (Q3) were used. For measurement data following a normal distribution and having homogeneous variances, the *t*-test was used; if not, the non-parametric rank-sum test was used, specifically the Mann-Whitney test for this study. The Kolmogorov-Smirnov test was used to ascertain the normal distribution of data and the ANOVA test was used to test the homogeneity of variances. The significance level was set at α = 0.05, with *P* < 0.05 indicating statistical significance.

#### 2.6.2 Propensity score matching

Matching was performed based on variables including Age, BMI, AMH, and AFC of the women. The caliper matching method was used with a caliper value set at 0.05, and a 1:3 matching ratio was applied.

#### 2.6.3 Collinearity diagnosis

Tolerance and Variance Inflation Factors were used to identify multicollinearity among independent variables. Pearson or Spearman rank correlation coefficient was used to determine the correlation between variables. When a strong correlation was observed between two variables, one was eliminated based on discussion by the research team.

#### 2.6.4 Logistic regression analysis

In this study, logistic regression analysis was used to evaluate the efficacy and safety of GnRH antagonists (Ganirelix and Cetrorelix) in IVF/ICSI treatment, and the odds ratio (or) and 95% confidence interval (CI) of relevant factors were calculated. First, univariate logistic regression was used to screen potential variables (α = 0.05) affecting the outcome of live birth, and variables were selected for multivariate analysis combined with clinical and statistical significance. “Whether live birth occurred” was the outcome indicator of the multivariate logistic regression. Akaike information criterion (AIC) and Bayesian information criterion (BIC) were used to evaluate the goodness of fit of the model, and the optimal multivariate logistic regression model was finally determined.

## 3 Results

### 3.1 Clinical characteristics

In this controlled experiment, propensity score matching was used for Age, BMI, AMH, and AFC, with a matching ratio of 1:3. Approximately 9,424 patients were included after matching, with 2,365 patients in the group using Cetrorelix (Group A), and 7,059 patients in the group using Ganirelix (Group B) as the antagonist. The baseline characteristics after matching are shown in Table 1.

**Table 1:** Comparison of basic characteristics. Median (P25, P75).

Characteristics	Before PSM				After PSM			
	Total (*n* = 11,491)	Group A (*n* = 2,731)	Group B (*n* = 9,120)	*p*	Total (*n* = 9,424)	Group A (*n* = 2,365)	Group B (*n* = 7,059)	*p*
Female age (years)	31.00 [28.00, 33.00]	31.00 [28.00, 33.00]	31.00 [28.00, 33.00]	0.400	31.00 [28.00, 34.00]	31.00 [28.00, 33.00]	31.00 [28.00, 34.00]	0.830
Age <35	9,518 (82.8%)	1,962 (82.7%)	7,556 (82.9%)	0.93	7,735 (82.1%)	1,957 (82.7%)	5,778 (81.9%)	0.341
Age ≥35	1,973 (17.2%)	409 (17.3%)	1,564 (17.1%)		1,689 (17.9%)	408 (17.3%)	1,281 (18.1%)	
BMI (kg/m^2^)	21.83 [19.98, 24.14]	22.04 [20.20, 24.41]	21.76 [19.95, 24.04]	0.010	22.03 [20.07, 24.25]	22.04 [20.20, 24.36]	22.03 [20.03, 24.23]	0.829
Underweight (<18.5)	1,067 (9.3%)	212 (8.9%)	855 (9.4%)	<0.001	807 (8.6%)	211 (8.9%)	596 (8.4%)	0.026
Normal weight (18.5 ≤ 24.0)	7,322 (63.7%)	1,455 (61.4%)	5,867 (64.3%)		5,948 (63.1%)	1,455 (61.5%)	4,493 (63.6%)	
Overweight (24.0 ≤ 28.0)	2,430 (21.1%)	579 (24.4%)	1,851 (20.3%)		2,128 (22.6%)	579 (24.5%)	1,549 (21.9%)	
Obesity (≥28.0)	672 (5.8%)	125 (5.3%)	547 (6.0%)		541 (5.7%)	120 (5.1%)	421 (6.0%)	
Gravidity (number of pregnancies)	1.00 [0.00, 2.00]	1.00 [0.00, 2.00]	1.00 [0.00, 2.00]	0.960	1.00 [0.00, 2.00]	1.00 [0.00, 2.00]	1.00 [0.00, 2.00]	0.547
Parity (number of births)	0.00 [0.00, 0.00]	0.00 [0.00, 0.00]	0.00 [0.00, 0.00]	0.040	0.00 [0.00, 0.00]	0.00 [0.00, 0.00]	0.00 [0.00, 0.00]	0.113
Infertile duration (years)	3.00 [2.00, 5.00]	3.00 [1.00, 5.00]	3.00 [2.00, 5.00]	0.790	3.00 [2.00, 5.00]	3.00 [1.00, 5.00]	3.00 [2.00, 5.00]	0.716
Infertility Duration ≥10 years (%)	552 (4.8%)	119 (5.0%)	433 (4.7%)	0.620	456 (4.8%)	119 (5.0%)	337 (4.8%)	0.653
Infertile type				0.427				
Primary infertility	5,616 (48.9%)	1,176 (49.6%)	4,440 (48.7%)	0.440	4,550 (48.3%)	1,173 (49.6%)	3,377 (47.8%)	0.145
Secondary infertility	5,875 (51.1%)	1,195 (50.4%)	4,680 (51.3%)		4,874 (51.7%)	1,192 (50.4%)	3,682 (52.2%)	
Infertile factors								
Tubal/pelvic factor	2,129 (18.5%)	432 (18.2%)	1,697 (18.6%)	0.690	1,762 (18.7%)	432 (18.3%)	1,330 (18.8%)	0.555
Polycystic ovary syndrome	321 (2.8%)	50 (2.1%)	271 (3.0%)	0.030	246 (2.6%)	50 (2.1%)	196 (2.8%)	0.094
Ovarian dysfunction	150 (1.3%)	30 (1.3%)	120 (1.3%)	0.930	131 (1.4%)	30 (1.3%)	101 (1.4%)	0.630
Insulin resistance	11 (0.1%)	2 (0.1%)	9 (0.1%)	>0.999	9 (0.1%)	2 (0.1%)	7 (0.1%)	>0.999
Advanced maternal age	1,973 (17.2%)	409 (17.3%)	1,564 (17.1%)	0.930	1,689 (17.9%)	408 (17.3%)	1,281 (18.1%)	0.341
Recurrent miscarriage	42 (0.4%)	10 (0.4%)	32 (0.4%)	0.750	35 (0.4%)	10 (0.4%)	25 (0.4%)	0.780
Recurrent IVF failure	42 (0.4%)	7 (0.3%)	35 (0.4%)	0.660	32 (0.3%)	7 (0.3%)	25 (0.4%)	0.828
Uterine pathology or structural abnormalities	326 (2.8%)	68 (2.9%)	258 (2.8%)	0.970	273 (2.9%)	68 (2.9%)	205 (2.9%)	0.999
Fertilization type								
IVF	9,811 (85.4%)	2,056 (86.7%)	7,755 (85%)	0.039	8,055 (85.5%)	2,050 (86.7%)	6,005 (85.1%)	0.059
ICSI	1,680 (14.6%)	315 (13.3%)	1,365 (15%)		1,369 (14.5%)	315 (13.3%)	1,054 (14.9%)	
AMH (μg/L)	4.87 [2.83, 7.27]	4.93 [2.86, 7.35]	4.60 [2.65, 6.95]	0.020	4.73 [2.71, 6.98]	4.59 [2.65, 6.95]	4.75 [2.71, 6.99]	0.968
AMH <1.1	506 (4.4%)	123 (5.2%)	383 (4.2%)	<0.001	453 (4.8%)	123 (5.2%)	330 (4.7%)	0.245
1.1 ≤AMH <4	3,966 (34.5%）	879 (37.1%)	3,087 (33.8%)		3,404 (36.1%)	877 (37.1%)	2,527 (35.8%)	
AMH ≥4	7,019 (61.1%)	1,369 (57.7%)	5,650 (62.0%)		5,567 (59.1%)	1,365 (57.7%)	4,202 (59.5%)	
AFC (*n*)	20.00 [13.00, 28.00]	20.00 [13.00, 28.00]	19.00 [13.00, 27.00]	0.060	20.00 [13.00, 27.00]	19.00 [13.00, 27.00]	20.00 [13.00, 27.00]	0.809
AFC <5	199 (1.7%)	37 (1.6%)	162 (1.8%)	0.115	169 (1.8%)	37 (1.6%)	132 (1.9%)	0.597
5 ≤AFC<15	3,194 (27.8%)	698 (29.4%)	2,496 (27.3%)		2,790 (29.6%)	696 (29.4%)	2,094 (29.7%)	
AFC ≥15	8,098 (70.5%)	1,636 (69.0%)	6,462 (70.9%)		6,465 (68.6%)	1,632 (69.0%)	4,833 (68.5%)	
Basal E2 level (pg/ml)	39.00 [29.00, 51.00]	45.00 [34.00, 57.00]	37.00 [29.00, 49.00]	<0.001	39.00 [29.80, 51.00]	45.00 [34.00, 57.00]	37.00 [28.05, 49.00]	<0.001
Basal FSH level (IU/L)	7.23 [6.24, 8.43]	7.21 [6.19, 8.43]	7.25 [6.24, 8.43]	0.270	7.26 [6.24, 8.44]	7.21 [6.18, 8.43]	7.28 [6.25, 8.46]	0.116
Basal LH level (mIU/ml)	4.41 [3.26, 6.12]	4.42 [3.26, 6.15]	4.41 [3.26, 6.11]	0.410	4.35 [3.22, 6.02]	4.42 [3.27, 6.15]	4.33 [3.21, 5.98]	0.557
Basal P level (ng/ml)	0.57 [0.38, 0.83]	0.60 [0.39, 0.84]	0.57 [0.37, 0.83]	0.069	0.58 [0.38, 0.83]	0.60 [0.39, 0.84]	0.57 [0.37, 0.83]	0.475
Gn starting dose (IU)	182.20 (50.52)	179.47 (49.94)	182.91 (50.64)	<0.001	187.50 [150.00, 225.00]	175.00 [150.00, 225.00]	187.50 [150.00, 225.00]	<0.001
150 IU (%)	3,955 (34.4%)	924 (39.0%)	3,031 (33.2%)	<0.001	3,147 (33.4%)	924 (39.1%)	2,223 (31.5%)	<0.001
225 IU (%)	4,151 (36.1%)	771 (32.5%)	3,380 (37.1%)		3,488 (37.0%)	766 (32.4%)	2,722 (38.6%)	
Other dosage (%)	3,385 (29.5%)	676 (28.5%)	2,709 (29.7%)		2,789 (29.6%)	675 (28.5%)	2,114 (29.9%)	
Recombinant FSH dose	1,739.67 (652.64)	1,739.95 (652.67)	1,738.57 (652.66)	0.930	1,752.81 (658.26)	1,738.28 (653.02)	1,757.68 (659.98)	0.212
Recombinant FSH duration	9.19 (2.10)	9.33 (1.86)	9.15 (2.15)	<0.001	9.20 (2.16)	9.34 (1.86)	9.16 (2.25)	<0.001
Recombinant LH dose	12.74 (52.51)	5.00 (32.00)	14.75 (56.47)	<0.001	12.74 (52.51	4.89 (30.76)	15.12 (56.23)	<0.001
Recombinant LH duration	0.17 (0.69)	0.06 (0.42)	0.20 (0.74)	<0.001	0.16 (0.69)	0.05 (0.38)	0.20 (0.74)	<0.001
FSH total dose	1,881.07 (905.464)	1,918.29 (970.796)	1,871.40 (887.493)	0.033	1,904.54 (922.32)	1,917.88 (971.66)	1,900.07 (905.22)	0.433
LH total dose	154.15 (467.35)	184.71 (541.88)	146.20 (445.62)	0.001	164.66 (484.91)	184.61 (541.97)	157.98 (464.08)	0.032
HMG total dose	141.40 (463.13)	179.71 (539.75)	131.44（440.52）	<0.001	151.73 (481.25)	179.60 (539.83)	142.39 (459.62)	0.003
HMG duration	0.92 (2.52)	1.12 (2.79)	0.87 (2.45)	<0.001	0.98 (2.60)	1.12 (2.79)	0.94 (2.53)	0.004
GnRH-A total dose		1.19 (0.38)	1.15 (0.36)	<0.001		1.19 (0.38)	1.14 (0.36)	<0.001
GnRH-A duration		4.77 (1.52)	4.60 (1.37)	<0.001		4.77 (1.52)	4.58 (1.39)	<0.001
GnRH-a dose on Trigger day	0.11 (0.09)	0.10 (2.79)	0.12 (0.09)	<0.001	0.11 (0.09)	0.10 (0.09)	0.11 (0.09)	<0.001
HCG dose on Trigger day	3,520.97 (2,390.79）	3,559.68 (2,298.99)	3,510.91 (2,414.11)	0.363	3,579.74 (2,370.04)	3,559.83 (2,299.74)	3,586.41 (2,393.25)	0.630
FSH level on Trigger day (IU/L)	12.67 [10.26, 15.95]	12.39 [10.09, 16.14]	12.72 [10.29, 15.90]	0.780	12.72 [10.32, 16.06]	12.39 [10.08, 16.14]	12.80 [10.37, 16.03]	0.228
LH level on trigger Day (mIU/ml)	2.41 [1.34, 4.17]	2.12 [1.22, 3.54]	2.50 [1.40, 4.37]	<0.001	2.40 [1.35, 4.13]	2.12 [1.22, 3.54]	2.50 [1.41, 4.37]	<0.001
E2 level on trigger day (pg/ml)	3,445.00 [1,923.00, 5,010.00]	3,545.00 [2,037.00, 5,010.00]	3,422.00 [1,891.00, 5,010.00]	0.011	3,366.50 [1,864.00, 5,010.00]	3,545.00 [2,037.00, 5,010.00]	3,305.00 [1,806.00, 5,010.00]	<0.001
*P* level on trigger day (ng/ml)	1.13 [0.72, 1.60]	1.11 [0.73, 1.57]	1.14 [0.72, 1.61]	0.500	1.13 [0.71, 1.60]	1.11 [0.73, 1.57]	1.13 [0.71, 1.61]	0.979
Trigger day LH ≧10 U/L	786 (6.8%)	117 (4.9%	669 (7.3%)	<0.001	650 (6.9%)	115 (4.9%)	535 (7.6%)	<0.001
Trigger day LH/Gn day LH ≥ 2	934 (8.1%)	147 (6.2%)	787 (8.6%)	<0.001	797 (8.5%)	145 (6.1%)	652 (9.2%)	<0.001
Endometrial morphology on hCG day								
A	7,020 (61.1%)	1,569 (66.2%)	5,451 (59.8%)	<0.001	5,806 (61.6%)	1,565 (66.2%)	4,241 (60.1%)	<0.001
B	2,868 (25.0%)	499 (21.0%)	2,369 (26.0%)		2,334 (24.8%)	497 (21.0%)	1,837 (26.0%)	
C	704 (6.1%)	126 (5.3%)	578 (6.3%)		573 (6.1%)	126 (5.3%)	447 (6.3%)	
ELSE	899 (7.8%)	177 (7.5%)	722 (7.9%)		711 (7.5%)	177 (7.5%)	534 (7.6%)	
Endometrial Thickness(mm)	9.00 [8.00, 11.00]	9.00 [8.00, 11.00]	9.00 [8.00, 11.00]	0.011	9.00 [8.00, 11.00]	9.00 [8.00, 11.00]	9.00 [8.00, 11.00]	0.010
Number of embryos for ET								
1 embryo	4,067 (35.4%)	817 (34.5%)	3,250 (35.6%)	0.296	3,353 (35.58%)	673 (28.46%)	2,680 (37.77%)	0.197
2 embryo	7,424 (64.6%)	1,554 (65.5%)	5,870 (64.4%)		6,121 (64.95%)	1,290 (54.95%)	4,831 (68.09%)	
Embryo stage								
D3	432 (3.8%)	102 (4.3%)	330 (3.6%)	0.192	354 (3.8%)	102 (4.3%)	252 (3.6%)	0.272
D5/D6	3,633 (31.6%)	715 (30.2%)	2,918 (32.0%)		2,932 (31.1%)	713 (30.1%)	2,219 (31.4%)	
D3 + D3	2,410 (21.0%)	522 (22.0%)	1,888 (20.7%)		2,008 (21.3%)	521 (22.0%)	1,487 (21.1%)	
D3 + D5/D6	142 (1.2%)	26 (1.1%)	116 (1.3%)		122 (1.3%)	25 (1.1%)	97 (1.4%)	
D5/D6 + D5/D6	4,870 (42.4%)	1,006 (42.4%)	3,864 (42.4%)		4,006 (42.5%)	1,004 (42.5%)	3,002 (42.5%)	
ELSE	4 (0.0%）	0	4 (0.0%）		2 (0.0%)	0 (0.0%)	2 (0.0%)	
Male information								
Male age	32.00 [29.00, 35.00]	32.00 [30.00, 36.00]	32.00 [29.00, 35.00]	0.050	32.00 [29.00, 35.00]	32.00 [30.00, 36.00]	32.00 [29.00, 35.00]	0.192
Abnormal semen analysis	6,803 (59.2%)	1,436 (60.6%)	5,367 (58.8%)	0.140	5,592 (59.3%)	1,431 (60.5%)	4,161 (58.9%)	0.189
DFI（%）	12.70 [8.55, 19.72]	12.67 [8.21, 19.72]	12.71 [8.61, 19.73]	0.680	12.80 [8.56, 19.74]	12.64 [8.18, 19.72]	12.85 [8.64, 19.76]	0.534
DFI<30	10,540 (91.7%)	2,167 (91.4%)	8,373 (91.8%)	0.540	8,645 (91.7%)	2,162 (91.4%)	6,483 (91.8%)	0.546
DFI≥30	951 (8.3%)	204 (8.6%)	747 (8.2%)		779 (8.3%)	203 (8.6%)	576 (8.2%)	
OHSS	113 (1.0%)	11 (0.4%)	102 (1.1%)	0.006	87 (0.9%)	10 (0.4%)	77 (1.1%)	0.010
Early-onset OHSS	73 (0.6%)	9 (0.3%)	64 (0.7%)	0.061	58 (0.6%)	8 (0.3%)	50 (0.7%)	0.060
Late-onset OHSS	40 (0.3%)	2 (0.1%)	38 (0.4%)		29 (0.3%)	2 (0.1%)	27 (0.4%)	
Degree of OHSS								
Mild	53 (0.5%)	4 (0.1%)	49 (0.5%)	0.091	43 (0.5%)	4 (0.2%)	39 (0.6%)	0.079
Moderate	36 (0.3%)	4 (0.1%)	32 (0.4%)		25 (0.3%)	4 (0.2%)	21 (0.3%)	
Severe	24 (0.2%)	3 (0.1%)	21 (0.2%)		19 (0.2%)	2 (0.1%)	17 (0.2%)	

PSM, propensity score matching; BMI, body mass index; IVF, *in vitro* fertilization; ICSI, intracytoplasmic sperm injection; AMH, anti-Mullerian hormone; FSH, follicular stimulating hormone; LH, luteinizing hormone; AFC, antral follicle counting; HMG, human menopausal gonadotropin; D3, cleavage-stage embryo; D5/D6/ELSE, blastocyst; OHSS, ovarian hyperstimulation syndrome.

No significant difference was observed in overall body weight between the groups (independent samples *t*-test, *p* = 0.829). However, the prevalence of obesity (BMI ≥ 28.0 kg/m^2^) was significantly higher in Group B (6.0%, 421/7,059) compared to Group A (5.1%, 120/2,365; *χ*^2^ test, *p* = 0.026), indicating a statistically elevated risk of obesity in Group B. The Gn starting dose during the ovarian stimulation cycle significantly differed between groups (Mann-Whitney *U* test, *p* < 0.001). Group A received a median dose of 175.00 IU (IQR 150.00–225.00 IU), while Group B had a higher median starting dose of 187.50 IU (IQR 150.00–225.00 IU). Furthermore, the proportion of patients requiring high-dose Gn regimens was significantly elevated in Group B (38.6%, 2,722/7,059) compared to Group A (32.4%, 766/2,365; *χ^2^* test, *p* < 0.001). Group A exhibited significantly higher values compared to Group B across three stimulation parameters: LH total dose (184.61 ± 541.97 IU) vs. IU (157.98 ± 464.08 IU; independent samples *t*-test, *p* = 0.032); HMG total dose (179.60 ± 539.83 IU) vs. (142.39 ± 459.62 IU; *t*-test, *p* = 0.003); HMG duration (1.12 ± 2.79 days) vs. (0.94 ± 2.53 days; *t*-test, *p* = 0.013). After reviewing the data, it was observed that the number of patients requiring recombinant LH and HMG in clinical practice is relatively small. As a result, the data obtained may be subject to considerable bias. Therefore, while the differences in LH total dose, HMG total dose, and HMG duration were found to be statistically significant, these values have limited clinical relevance within the context of this trial.

Significant differences were observed in hormonal profiles on the trigger day. Group A demonstrated a median of 2.12 mIU/ml (IQR 1.22–3.54), significantly lower than Group B (median 2.50 mIU/ml, IQR 1.41–4.37; Mann-Whitney *U* test, *p* < 0.001) on LH level. Group A exhibited higher median concentrations (3,545.00 pg/ml, IQR 2,037.00–5,010.00) compared to Group B (3,305.00 pg/ml, IQR 1,806.00–5,010.00; Mann-Whitney *U* test, *p* < 0.001) on E2 level.

The effectiveness of GnRH antagonists in preventing premature follicular rupture was robustly demonstrated. Group A demonstrated superior performance compared to Group B. On the aspect of LH ≥10 U/L on trigger day, the incidence was significantly lower in Group A (4.9%, 115/2,365) than in Group B (7.6%, 535/7,059, *p* < 0.001). On the aspect of LH ratio (trigger day/Gn day) ≥2, Group A showed a lower proportion (6.1%, 145/2,365) compared to Group B (9.2%, 652/7,059; *χ*² test, *p* < 0.001).

A statistically significant difference in endometrial morphology was observed between the groups. Group A demonstrated a more favorable endometrial receptivity profile, with 66.2% (1,565/2,365) of patients exhibiting optimal Type A morphology compared to 60.1% (4,241/7,059) in Group B. Conversely, the proportion of suboptimal Type C morphology was lower in Group A (5.3%, 126/2,365) than in Group B (6.3%, 447/7,059). These findings collectively indicate that Group A exhibited superior endometrial morphological characteristics, potentially associated with enhanced receptivity outcomes.

Furthermore, The incidence of ovarian hyperstimulation syndrome (OHSS) significantly differed between the groups, with Group B demonstrating a higher overall rate (1.1%, 77/7,059) compared to Group A (0.4%, 10/2,365; *p* = 0.01). Subgroup analysis of OHSS onset timing revealed no statistically significant differences in early-onset OHSS [Group A: 0.3% (8/2,365) vs. Group B: 0.7% (50/7,059); *p* = 0.06], whereas late-onset OHSS exhibited a numerically elevated proportion in Group B [0.4% (27/7,059) vs. Group A: 0.1% (2/2,365)], though this difference did not reach statistical significance (*p* = 0.010).The incidence rates of OHSS between the groups did not differ significantly. Detailed results can be found in Table 2.

**Table 2:** Embryo status and pregnancy outcomes-median (P25, P75).

Characteristics	Before PSM				After PSM			
	Total (*n* = 11,491)	Group A (*n* = 2,371)	Group B (*n* = 9,120)	*p*	Total (*n* = 9,424)	Group A (*n* = 2,365)	Group B (*n* = 7,059)	*p*
Overall pregnancy status								
Live birth	5,563 (48.4%)	1,119 (47.2%)	4,444 (48.7%)	0.191	4,603 (48.8%)	1,117 (47.2%)	3,486 (49.4%)	0.074
Premature birth	2,040 (17.8%)	417 (17.6%)	1,623 (17.8%)	0.573	1,675 (17.8%)	416 (17.6%)	1,259 (17.8%)	0.240
Full-term delivery	3,494 (30.4%)	696 (29.4%)	2,798 (30.7%)	0.575	2,902 (30.8%)	695 (29.4%)	2,207 (31.3%)	0.290
Biochemical pregnancy (14 day)	7,754 (67.5%)	1,581 (66.7%)	6,173 (67.7%)	0.365	6,386 (67.8%)	1,576 (66.6%)	4,810 (68.1%)	0.185
Clinical pregnancy (28 days)	6,365 (55.4%)	1,300 (54.8%)	5,065 (55.5%)	0.552	5,263 (55.8%)	1,296 (54.8%)	3,967 (56.2%)	0.245
Miscarriage	2,192 (19.07%)	463 (19.5%)	1,729 (19.0%)	0.841	1,783 (18.9%)	459 (19.4%)	1,324 (18.8%)	0.862
Fresh embryo transfer								
Live birth	1,103 (41.3%)	218 (38.3%)	893 (42.1%)	0.107	979 (43.4%)	217 (38.4%）	762 (45.2%)	0.107
Biochemical pregnancy (14 days)	1,565 (58.1%)	317 (55.7%)	1,248 (58.8%)	0.187	1,346 (59.8%)	316 (55.9%）	1,030 (58.1%)	0.097
Clinical pregnancy (28 days)	1,281 (47.6%)	256 (45.0%)	1,025 (48.3%)	0.163	1,123 (49.8%）	258 (45.7%）	865 (49.3%)	0.163
Miscarriage	454 (16.8%)	99 (17.4%)	355 (16.7%)	0.562	396 (17.5%)	99 (17.5%)	297 (17.6%)	0.834
Fresh embryo transfer rate (%)	23.42	24	23.27	0.317	23.38	23.27	23.4	0.532
Frozen embryo transfer (FET)								
Live birth	4,425 (50.2%)	901 (50.0%)	3,551 (50.8%)	0.654	3,624 (50.5%)	900 (50.0%）	2,724 (50.6%)	0.654
Biochemical Pregnancy (14 days)	6,189 (70.3%)	1,264 (70.1%)	4,925 (70.4%)	0.840	5,040 (70.2%)	1,260 (70.0%）	3,780 (70.3%)	0.824
Clinical Pregnancy (28 days)	5,084 (57.8%)	1,044 (57.9%)	4,040 (57.7%)	0.880	4,140 (57.7%)	1,038 (57.7%）	3,102 (57.7%)	0.874
Miscarriage	1,738 (19.6%)	364 (20.1%)	1,374 (19.6%)	0.991	1,387 (19.3%)	360 (20.0%)	1,027 (19.1%)	0.762
Oocyte and embryo status								
MII oocytes (mature eggs)	12.00 (8.00, 17.00)	12.00 (8.00, 17.00)	12.00 (8.00, 17.00)	0.726	12.00 (8.00, 17.00)	12.00 (8.00, 17.00)	12.00 (8.00, 17.00)	0.059
Number of 2PN embryo(s)	9.00 (5.00, 13.00)	9.00 (6.00, 13.00)	9.00 (5.00, 13.00)	0.231	9.00 (5.00, 13.00)	9.00 (6.00, 13.00)	8.00 (5.00, 13.00)	0.011
Number of usable Embryos (D3)	8.00 (4.00, 12.00)	7.00 (4.00, 12.00)	8.00 (4.00, 12.00)	0.909	7.00 (4.00, 12.00)	7.00 (4.00, 12.00)	7.00 (4.00, 12.00)	0.143
Cleavage-stage Embryos of good quality	3.00 (1.00, 6.00)	3.00 (1.00, 6.00)	3.00 (1.00, 6.00)	0.190	3.00 (1.00, 6.00)	3.00 (1.00, 6.00)	3.00 (1.00, 6.00)	0.300
Blastocysts of good quality	0.00 [0.00, 0.00]	0.00 [0.00, 0.00]	0.00 [0.00, 0.00]	<0.001	0.00 [0.00, 0.00]	0.00 [0.00, 0.00]	0.00 [0.00, 0.00]	<0.001
Oocyte fertilization rate (%)	78.57 (66.67, 87.50)	77.78 (66.67) 87.50)	80.00 (66.67, 87.50)	0.012	78.89 (66.67, 87.91)	77.78 (66.67) 87.55)	79.98 (66.67, 87.43)	0.094

### 3.2 Embryo outcomes and pregnancy outcomes

Live birth and clinical pregnancy rates demonstrated comparable patterns between groups, with Group B consistently showing numerically higher-though statistically nonsignificant-values across all cycle types. In the overall analysis, Group A achieved a live birth rate of 47.2% (1,117/2,365) vs. 49.4% (3,486/7,059) in Group B (*χ*^2^ test, *p* = 0.074), while clinical pregnancy rates were 54.8% (1,296/2,365) in Group A compared to 56.2% (3,967/7,059) in Group B (*p* = 0.245). Subgroup analyses for live births revealed rates of 38.4% (217/565) in Group A vs. 45.2% (762/1,685) in Group B (*p* = 0.107) for fresh embryo transfers, and 50.0% (900/1,800) vs. 50.7% (2,724/5,374) (*p* = 0.654) for frozen-thawed embryo transfer (FET) cycles. Similarly, clinical pregnancy rates in fresh transfers were 45.7% (258/565) for Group A vs. 49.29% (865/1,685) for Group B (*p* = 0.163), with FET cycles showing nearly identical rates of 57.7% (1,038/1,800) in Group A and 57.70% (3,102/5,374) in Group B (*p* = 0.874).

A significant intergroup difference was observed in the number of 2PN embryos, with Group A demonstrating a higher median count [9.00 (IQR 6.00–13.00)] compared to Group B [8.00 (IQR 5.00–13.00); Mann-Whitney *U* test, *p* = 0.011]. While statistically significant, the modest numerical disparity (median difference = 1.00) suggests limited clinical relevance, warranting cautious interpretation in the context of overall embryological outcomes. No significant difference was observed in the number of good-quality cleavage-stage embryos between the two groups. Both Group A and Group B demonstrated comparable median values of 3.00 good-quality embryos (interquartile range 1.00–6.00 for each group; Mann-Whitney *U* test, *p* = 0.300), indicating statistically equivalent embryological quality at the cleavage stage. Despite a statistically significant difference in Blastocysts of good quality between groups [Group A: median 0.00 (IQR 0.00–0.00) vs. Group B: 0.00 (0.00–0.00), *p* < 0.001], this metric lacks clinical interpretability due to insufficient data volume in both cohorts, resulting in skewed distributions and unreliable statistical comparisons. Consequently, this outcome should be interpreted with caution and excluded from definitive conclusions.

### 3.3 Univariate logistic regression analysis and multivariate logistic regression analysis

Univariate logistic regression analysis revealed significant associations between multiple baseline/cycle characteristics and clinical outcomes in the 1:3 matched cohort. The results can be found in Table 3. Key demographic predictors included female age ≥35 years (OR = 0.52, 95% CI 0.46–0.58, *p* < 0.001) and secondary infertility (OR = 0.88, 95% CI 0.82–0.96, *p* = 0.003), both demonstrating reduced odds of the primary outcome. Ovarian reserve markers showed strong dose-response relationships: AMH ≥ 4 μg/L (OR = 1.87, 95% CI 1.54–2.28, *p* < 0.001) and AFC ≥ 15 (OR = 1.77, 95% CI 1.30–2.44, *p* < 0.001) were associated with increased success probabilities.

Stimulation parameters exhibited mixed effects—Gn starting dose of 150 IU increased odds (OR = 1.25, 95% CI 1.13–1.39, *p* < 0.001), whereas prolonged HMG duration reduced likelihood (OR = 0.96/day, 95% CI 0.95–0.98, *p* < 0.001). Trigger-day biomarkers demonstrated critical associations: each 1,000 pg/ml increase in E2 level amplified odds (OR = 1.00, *p* < 0.001), while elevated FSH (OR = 0.98/IU, *p* < 0.001) and progesterone (OR = 1.15/ng/ml, *p* < 0.001) showed paradoxical effects.

**Table 3:** (1:3 matching for categorical variables) univariate logistic regression analysis.

Characteristics	*β*	SE	Wald	OR	95%CI		*P*
					Lower limit	Upper	
Female age (years)							
Age <35	1 (reference)						
Age ≥35	−0.66	0.056	−11.856	0.517	0.463	0.576	**<0**.**001**
BMI (kg/m^2^)							
Underweight (<18.5)	1 (reference)						
Normal weight (18.5 ≤24.0)	0.03	0.075	0.403	1.031	0.890	1.194	0.687
Overweight (24.0 ≤28.0)	−0.024	0.051	−0.483	0.976	0.884	1.078	0.629
Obesity (≥28.0)	−0.099	0.09	−1.104	0.905	0.759	1.080	0.270
Gravidity (number of pregnancies)	−0.087	0.016	−5.431	0.917	0.889	0.946	**<0**.**001**
Parity (number of births)	−0.354	0.05	−7.035	0.702	0.636	0.774	**<0**.**001**
Infertility duration ≥10 years (%)	−0.276	0.097	−2.847	0.758	0.626	0.917	**0**.**004**
Infertile type							
Primary infertility	1 (reference)						
Secondary infertility	−0.124	0.041	−2.995	0.884	0.815	0.958	**0**.**003**
Infertile factors							
Tubal/pelvic factor	−0.382	0.054	−7.145	0.682	0.614	0.757	**<0**.**001**
Polycystic ovary syndrome	−0.17	0.13	−1.311	0.843	0.653	1.087	0.190
Ovarian dysfunction	−0.644	0.186	−3.46	0.525	0.362	0.752	**0**.**001**
Insulin resistance	−0.647	0.707	−0.916	0.523	0.110	1.985	0.360
Advanced maternal age	−0.66	0.056	−11.856	0.517	0.463	0.576	**<0**.**001**
Recurrent miscarriage	−0.126	0.34	−0.371	0.882	0.447	1.716	0.711
Recurrent IVF failure	−0.466	0.366	−1.275	0.627	0.298	1.267	0.202
Uterine pathology or structural abnormalities	−0.484	0.127	−3.817	0.616	0.479	0.788	**<0**.**001**
Fertilization type							
IVF	1 (reference)						
ICSI	−0.388	0.049	−7.93	0.679	0.617	0.747	**<0**.**001**
AMH (μg/L)							
AMH <1.1	1 (reference)						
1.1 ≤AMH <4	0.283	0.103	2.745	1.327	1.086	1.627	**0**.**006**
AMH ≥4	0.626	0.101	6.21	1.870	1.537	2.282	**<0**.**001**
AFC(n)							
AFC <5	1 (reference)						
5 ≤AFC <15	0.275	0.164	1.683	1.317	0.959	1.823	0.092
AFC ≥15	0.571	0.161	3.549	1.771	1.296	2.439	**<0**.**001**
Basal E2 level (pg/ml)	−0.001	0.001	−1.44	0.999	0.998	1.000	0.150
Basal FSH level (IU/L)	0.151	0.048	3.148	1.163	1.061	1.279	**0**.**002**
Basal LH level (mIU/ml)	0.033	0.007	4.792	1.033	1.020	1.047	**<0**.**001**
Basal P level (ng/ml)	−0.037	0.011	−3.293	0.963	0.942	0.985	**0**.**001**
Gn starting dose							
Other dosage (%）	1 (reference)						
150 IU (%)	0.225	0.052	4.319	1.252	1.131	1.387	**<0**.**001**
225 IU (%)	0	0.051	−0.007	1.000	0.905	1.105	0.994
Recombinant FSH dose	0	0	−2.166	1.000	1.000	1.000	**0**.**030**
Recombinant FSH duration	0.008	0.01	0.872	1.008	0.990	1.028	0.383
Recombinant LH dose	0	0	−0.614	1.000	0.999	1.001	0.539
Recombinant LH duration	−0.02	0.03	−0.663	0.980	0.925	1.039	0.507
FSH total dose	0	0	−4.305	1.000	1.000	1.000	**<0**.**001**
LH total dose	0	0	−5.279	1.000	1.000	1.000	**<0**.**001**
HMG total dose	0	0	−5.254	1.000	1.000	1.000	**<0**.**001**
HMG duration	−0.039	0.008	−4.8	0.962	0.947	0.977	**<0**.**001**
GnRH-A							
Ganirelix	1 (reference)						
Cetrorelix	−0.086	0.048	−1.813	0.917	0.836	1.007	0.070
Ganirelix total dose	0.061	0.035	1.732	1.063	0.992	1.139	0.083
Ganirelix duration	0.016	0.009	1.761	1.016	0.998	1.034	0.078
Cetrorelix total dose	−0.061	0.038	−1.617	0.941	0.874	1.013	0.106
Cetrorelix duration	−0.016	0.009	−1.66	0.985	0.967	1.003	0.097
GnRH-a dose on trigger day	1.02	0.235	4.342	2.772	1.750	4.394	**<0**.**001**
HCG dose on trigger day	0	0	−5.217	1.000	1.000	1.000	**<0**.**001**
E2 level on trigger day (pg/ml)	0	0	7.128	1.000	1.000	1.000	**<0**.**001**
FSH level on trigger day (IU/L)	−0.019	0.004	−4.35	0.981	0.973	0.990	**<0**.**001**
LH level on trigger day (mIU/ml)	−0.004	0.003	−1.215	0.996	0.991	1.002	0.224
*P* level on trigger day (ng/ml)	0.141	0.026	5.497	1.151	1.095	1.211	**<0**.**001**
Trigger day LH ≧10 U/L	−0.063	0.081	−0.771	0.939	0.800	1.101	0.441
Trigger day LH/Gn Day LH ≥ 2	−0.161	0.074	−2.167	0.851	0.735	0.984	**0**.**030**
Endometrial morphology on hCG day							
A	1 (reference)						
B	0.072	0.049	1.467	1.075	0.976	1.183	0.142
C	−0.129	0.088	−1.467	0.879	0.739	1.044	0.142
ELSE	0.003	0.079	0.038	1.003	0.858	1.172	0.969
Endometrial thickness(mm)	0.021	0.011	1.919	1.022	1.000	1.044	0.055
Number of embryos for ET							
1 embryo	1 (reference)						
2 embryo	0.419	0.044	9.598	1.520	1.395	1.655	**<0**.**001**
Embryo stage							
D3	1 (reference)						
D5/D6	0.741	0.129	5.748	2.098	1.636	2.712	**<0**.**001**
D3 + D3	1.032	0.218	4.741	2.806	1.833	4.308	**<0**.**001**
D3 + D5/D6	0.791	0.126	6.257	2.205	1.728	2.837	**<0**.**001**
D5/D6 + D5/D6	1.331	0.125	10.652	3.784	2.973	4.855	**<0**.**001**
ELSE	−10.534	139.277	−0.076	0.000		83,305.302	0.940
Male information							
Male age	−0.037	0.004	−8.884	0.964	0.956	0.972	**<0**.**001**
Abnormal semen analysis	−0.038	0.042	−0.895	0.963	0.887	1.046	0.371
DFI (%)							
DFI <30	1 (reference)						
DFI ≥30	−0.121	0.075	−1.607	0.886	0.765	1.027	0.108
Degree of OHSS							
NO OHSS	1 (reference)						
Mild	0.454	0.913	0.498	1.575	0.261	11.965	0.619
Moderate	0.049	0.5	0.098	1.050	0.386	2.856	0.922
Severe	1.371	0.563	2.436	3.938	1.428	13.825	**0**.**676**

PSM, propensity score matching; BMI, body mass index; IVF, *in vitro* fertilization; ICSI, intracytoplasmic sperm injection.; AMH, anti-Mullerian hormone; FSH, follicular stimulating hormone; LH, luteinizing hormone; AFC, antral follicle counting; HMG, human menopausal gonadotropin; D3, cleavage-stage embryo; D5/D6/ELSE, blastocyst; OHSS, ovarian hyperstimulation syndrome.

The bold *P* values indicate statistical significance in univariate regression analysis with “whether live birth occurred”.

Embryological factors proved decisive, with dual embryo transfer (OR = 1.52 vs. single, *p* < 0.001) and blastocyst-stage embryos (D5/D6: OR = 2.10, *p* < 0.001) substantially improving outcomes. Male partners' age ≥40 years significantly diminished success (OR = 0.96/year, *p* < 0.001). Notably, BMI categories, PCOS status, and DFI ≥30% showed no significant associations (*p* > 0.05).

Multivariate logistic regression analysis identified several independent predictors of clinical outcomes after adjusting for confounders. Using ganirelix as the reference group, the adjusted odds ratio (aOR) for cetrorelix was 0.913 (95% CI: 0.83–1.01, *P* = 0.065). This result suggests that the live birth rate in the cetrorelix group may be slightly lower compared to ganirelix (OR < 1), but the difference did not reach statistical significance (*P* > 0.05). Although the confidence interval includes the null value (1.0), indicating limited clinical divergence in live birth outcomes between groups.

Advanced female age (≥35 years) significantly reduced success odds (adjusted OR = 0.65, 95% CI 0.57–0.74, *p* < 0.001), while each additional gravidity further decreased the likelihood by 16% (aOR = 0.84, 95% CI 0.74–0.94, *p* = 0.003). Ovarian reserve markers revealed AMH ≥ 4 μg/L as a positive predictor (aOR = 1.29, 95% CI 1.02–1.64, *p* = 0.034), though AFC categories lost significance (*p* > 0.05). Stimulation protocols showed dose-dependent benefits: Gn starting doses of 150 IU (aOR = 1.18, 95% CI 1.06–1.31, *p* = 0.004) and 225 IU (aOR = 1.15, 95% CI 1.02–1.30, *p* = 0.026) outperformed other regimens, whereas ICSI yielded lower success than IVF (aOR = 0.68, 95% CI 0.61–0.76, *p* < 0.001). Trigger-day progesterone elevation retained clinical relevance (aOR = 1.06 per ng/ml, 95% CI 1.00–1.12, *p* = 0.047). Embryologically, dual embryo transfer nearly doubled success rates vs. single transfers (aOR = 1.51, 95% CI 1.38–1.65, *p* < 0.001).

## 4 Discussion

GnRH antagonist protocols have become the mainstream regimen in controlled ovarian hyperstimulation, offering distinct advantages over agonist protocols. These include shorter ovarian stimulation cycles and improved time efficiency, making them suitable for most patient populations (9, 10).

Pharmacokinetically, Cetrorelix [0.25 mg subcutaneous (SC), 85% bioavailability, 6–8 h half-life] sustains LH suppression for 24 h post-discontinuation, though its efficacy in inhibiting E2 lacks consistent quantification (11). Ganirelix (0.25 mg SC, 91% bioavailability, 12–15 h half-life) allows hormonal recovery within 48 h while reducing E2 levels by 25% at standard dosing (12). The selective LH/follicle-stimulating hormone (FSH) suppression (>10:1 ratio) of these antagonists (13, 14) preserves follicular synchronization and supports GnRH agonist triggering, reducing moderate-to-severe OHSS incidence by 62% [odds ratio (OR) = 0.38] (15). This benefit is especially significant in high responders, where OHSS risk decreases from 23.4% to 6.8% with ≥15 oocytes retrieved (14). A statistically significant difference in trigger-day estradiol (E2) levels was observed between groups, with Group B (Ganirelix) demonstrating lower median concentrations [3,305.00 pg/ml (IQR 1,806.00–5,010.00)] compared to Group A [Cetrorelix: 3,545.00 pg/ml (2,037.00–5,010.00); *p* < 0.001]. This disparity may reflect Ganirelix's enhanced short-term estrogen suppression efficiency, as evidenced by its pharmacodynamic profile favoring transient E2 modulation. However, this hypothesis requires validation through dedicated pharmacodynamic studies controlling for ovarian response heterogeneity.

A significant disparity in ovarian hyperstimulation syndrome (OHSS) incidence was observed between the protocols, with Group B (Ganirelix) exhibiting a higher overall rate (1.1%, 77/7,059) compared to Group A (Cetrorelix: 0.4%, 10/2,365; *p* = 0.010). While early-onset OHSS showed no statistical difference [Group A: 0.3% (8/2,365) vs. Group B: 0.7% (50/7,059); *p* = 0.06], late-onset OHSS was numerically elevated in Group B [0.4% (27/7,059) vs. 0.1% (2/2,365); *p* = 0.15], aligning with Zhang et al.'s findings of increased severe OHSS risk with Ganirelix (*p* = 0.006) (16). Despite some studies reporting comparable OHSS rates [Ganirelix: 8.0% (7/87) vs. Cetrorelix: 6.8% (6/88)] (17) and similar trigger-day E2 levels/oocyte yields (18), Cetrorelix consistently demonstrated lower severe OHSS incidence [0.6% in literature (19); 0.084% in our trial], potentially attributable to its shorter half-life and transient LH suppression (18). These discrepancies may reflect population heterogeneity or protocol variations, but collectively underscore the need for heightened vigilance with Ganirelix, particularly in high responders, despite its equivalent efficacy in follicular synchronization and embryo outcomes.

In infertile women undergoing gonadotropin therapy, the development of multiple follicles can elevate estradiol (E2) to supraphysiological levels, potentially triggering an LH surge before follicle maturation. This phenomenon, termed “LH escape” by some scholars, is characterized by LH levels exceeding 10 U/L or rising 2–3 times above baseline before the trigger day. The likely mechanism involves heightened pituitary responsiveness to endogenous gonadotropin due to increasing estrogen levels (20). This premature LH surge can lead to luteinization of immature follicles, arresting oocyte development and necessitating cycle cancellation (17). Notably, “LH escape” is more frequent in patients of advanced age or those with diminished ovarian reserve (21). Antagonists achieve immediate pituitary suppression through competitive binding to GnRH receptors, demonstrating a 9-fold higher affinity compared to endogenous GnRH (13, 14, 22, 23). This pharmacological action enables significant LH reduction (<10 mIU/ml within 2–6 h) while maintaining premature LH surge rates below 1% (13, 14), effectively circumventing the initial stimulatory phase (“flare-up effect”) associated with agonist protocols (17). The absence of transient gonadotropin stimulation not only enhances pituitary safety but also translates to superior clinical outcomes, with studies demonstrating a 67% reduction in cycle cancellation rates compared to traditional agonist regimens (24).

Our study robustly demonstrated the effectiveness of GnRH antagonists in preventing premature follicular rupture, with Group A (receiving Cetrorelix) showing superior performance compared to Group B. Specifically, the incidence of luteinizing hormone (LH) levels ≥10 U/L on the trigger day was significantly lower in Group A (4.9%, 115/2,365) than in Group B (7.6%, 535/7,059; *p* < 0.001). Similarly, the proportion of patients with an LH ratio (trigger day/Gn day) ≥2 was lower in Group A (6.1%, 145/2,365) compared to Group B (9.2%, 652/7,059; *χ*² test, *p* < 0.001). These results are consistent with Zhang et al. (16), who reported no significant difference in spontaneous ovulation rates between the Ganirelix group and a comparator (0.6% vs. 0.6%, *p* > 0.05), reinforcing the reliability of GnRH antagonists. Literature further supports their efficacy, with no premature LH surges observed in antagonist-treated groups (17), and studies highlighting the comparable performance of Cetrorelix and Ganirelix in preventing premature ovulation (25). Beyond avoiding cycle cancellation, suppressing premature LH surges has broader implications. Elevated progesterone levels on the trigger day, often due to early LH rises, can advance endometrial maturation and alter gene expression, risking implantation failure (26). Additionally, an early LH surge may trigger luteinization of immature follicles, activating luteal cell pathways and increasing progesterone secretion (27). By effectively controlling LH levels, GnRH antagonists optimize the endometrial environment and prevent premature follicular luteinization, enhancing embryo implantation and development.

The interplay between estrogen and progesterone plays a pivotal role in endometrial development during IVF stimulation cycles, with hormonal imbalances significantly influencing embryo implantation outcomes. Studies have demonstrated that early progesterone elevation, as confirmed by histological and ultrastructural changes, induces premature endometrial maturation during the follicular phase (26). This effect is amplified in the late follicular phase, where elevated serum progesterone levels can trigger early endometrial development. Concurrently, higher estradiol (E2) levels in this phase enhance the upregulation of endometrial progesterone receptors, rendering the endometrium highly sensitive to even slight progesterone increases (26). Such premature development creates a discordance between endometrial stroma and glands, which negatively impacts embryo implantation (28). In our study, a statistically significant difference in endometrial morphology was observed. Group A exhibited a higher proportion of optimal Type A morphology (66.2%, 1,565/2,365) compared to Group B (60.1%, 4,241/7,059), while the suboptimal Type C morphology was less frequent in Group A (5.3%, 126/2,365) than in Group B (6.3%, 447/7,059). These findings indicate that Group A achieved a more favorable endometrial receptivity profile, potentially attributable to the use of Cetrorelix. This GnRH antagonist may more effectively suppress premature LH surges, thereby reducing progesterone's influence on the endometrium and optimizing morphological conditions for implantation.

This observation elucidates why fresh embryo transfers demonstrate lower rates of biochemical pregnancy (assessed at 14 days), clinical pregnancy (assessed at 28 days), and live birth compared to frozen-thawed embryo transfers. In fresh embryo transfer cycles, hormone levels—notably estrogen and progesterone—are frequently supraphysiological, meaning they exceed normal physiological ranges. These elevated levels can negatively impact endometrial receptivity, thereby reducing the likelihood of successful embryo implantation. Conversely, frozen-thawed embryo transfers are typically conducted in a more controlled hormonal environment, where hormone levels are often closer to physiological norms. This optimized hormonal milieu enhances endometrial conditions, leading to improved pregnancy outcomes.

For patients, live birth rate and clinical pregnancy rate are the primary indicators of concern. If the medications used during ovarian stimulation may affect the final pregnancy outcomes, it could have an adverse impact on the patients. Therefore, we focused on analyzing pregnancy-related metrics to ensure the safety and efficacy of the treatment protocols. Our findings indicate that Group A and Group B had comparable overall live birth rates and clinical pregnancy rates, with no statistically significant differences. Specifically, the overall analysis showed a live birth rate of 47.2% (1,117/2,365) for Group A and 49.4% (3,486/7,059) for Group B (*χ*² test, *p* = 0.074); clinical pregnancy rates were 54.8% (1,296/2,365) for Group A and 56.2% (3,967/7,059) for Group B (*p* = 0.245). This finding is consistent with the conclusion of Mingzhu Cao et al., who also reported no significant difference in embryo outcomes between the two groups (29).

In the fresh embryo transfer subgroup, Group B showed numerical advantages in live birth rate and clinical pregnancy rate, although these differences did not reach statistical significance. The live birth rate for Group A was 38.4% (217/565), and for Group B it was 45.2% (762/1,685) (*p* = 0.107); clinical pregnancy rates were 45.7% (258/565) for Group A and 49.29% (865/1,685) for Group B (*p* = 0.163). This trend is consistent with the findings of John et al., who observed a slightly higher live birth rate in the Ganirelix group [51.7% (45/87) vs. 48.9% (43/88)] (17). However, in frozen-thawed embryo transfer (FET) cycles, the results between the two groups were almost identical: the live birth rate for Group A was 50.0% (900/1,800), and for Group B it was 50.7% (2,724/5,374) (*p* = 0.654), while clinical pregnancy rates were both 57.7% (Group A: 1,038/1,800; Group B: 3,102/5,374; *p* = 0.874). These data suggest that the efficacy of the two antagonists is highly similar in FET cycles.

However, there remains heterogeneity in the existing evidence regarding the efficacy of the two antagonists. Check et al. reported that Ganirelix may be associated with a lower embryo implantation rate compared to Cetrorelix (30), but our trial and other studies have shown comparable outcomes between the two antagonist protocols (16). For example, one study indicated no significant difference in clinical pregnancy rates (47.7% vs. 45.9%) and live birth rates (37.5% vs. 33.6%) between Cetrorelix and Ganirelix (16). However, another study suggested that sustained pregnancy rates may favor Cetrorelix [35.1% (71/202) vs. 29.0% (91/313)] (31). Nevertheless, our results do not support this difference and instead show that the two antagonists perform similarly in terms of pregnancy outcomes.

While no significant intergroup difference in overall body weight was detected (*p* = 0.829), Group B demonstrated a clinically meaningful elevation in obesity prevalence (BMI ≥28.0 kg/m^2^: 6.0% vs. 5.1%, *p* = 0.026). Obesity may compromise GnRH antagonist efficacy through altered ovarian responsiveness—obese patients often require higher gonadotropin doses to achieve comparable oocyte yields (32) and exhibit 18%–22% lower viable embryo rates vs. normal-BMI counterparts (33). The elevated obesity burden in Group B could partially explain observed variations in cycle outcomes, particularly given adiposity-related pharmacokinetic alterations that reduce antagonist bioavailability (33). Critically, this adipose-mediated pharmacokinetic interference likely contributed to the significantly higher Gn starting doses in Group B (median 187.50 IU vs. 175.00 IU, *p* < 0.001), as increased adiposity may necessitate dose escalation to overcome reduced drug sensitivity (34).

Furthermore, no clear adverse events related to these two medications were reported in our study. Even when we reviewed the initial data collection phase, out of 51,869 cases, there was only one report of a local rash after the Ganirelix injection. This case was later excluded during the data cleaning stage because it did not meet the inclusion criteria. However, literature reports clinical data indicating that Cetrorelix and Ganirelix are well-tolerated. Common injection site reactions include redness, itching, and swelling, which are generally mild (23). The trial by John et al. found that the most common adverse event in both treatment groups was bloating, occurring in 10 out of 87 patients (11.5%) in Group B and 11 out of 88 patients (12.5%) in Group A. Approximately 10 patients reported injection site reactions to the antagonists (6 in the Group B and 4 in the Group A), all of which were mild (17).

The multivariate logistic regression analysis identified several independent predictors of live birth success while adjusting for key confounders (Table 4). Female age ≥35 years emerged as a strong negative predictor (aOR = 0.65, 95% CI 0.57–0.74, *p* < 0.001), consistent with established evidence that advanced maternal age compromises oocyte quality and endometrial receptivity (29). Notably, ICSI fertilization was associated with reduced live birth odds compared to conventional IVF (aOR = 0.68, 95% CI 0.61–0.76, *p* < 0.001), potentially reflecting sperm-related epigenetic modifications or technical variations in ICSI protocols (16).

**Table 4:** (1: 3 matching for categorical variables) multivariate logistic regression analysis.

Characteristics	*β*	SE	Wald	OR	95%CI		*P*
					Lower limit	Upper	
Intercept	−0.410	0.299	−1.374	0.663	0.369	1.189	0.169
Female age (years)							
Age <35	1 (reference)						
Age ≥35	−0.432	0.068	−6.376	0.649	0.568	0.741	<0.001
Gravidity (number of pregnancies)	−0.177	0.06	−2.939	0.838	0.744	0.942	0.003
Parity (number of births)	−0.003	0.021	−0.141	0.997	0.957	1.038	0.888
Infertility duration ≥10 years (%)	−0.059	0.102	−0.575	0.943	0.771	1.152	0.565
Infertile type							
Primary infertility	1 (reference)						
Secondary infertility	0.013	0.048	0.28	1.013	0.923	1.113	0.779
Infertile factors							
Ovarian dysfunction	0.053	0.21	0.254	1.055	0.695	1.584	0.800
Uterine pathology or structural Abnormalities	0.041	0.186	0.218	1.041	0.719	1.493	0.828
Fertilization type							
IVF	1 (reference)						
ICSI	−0.383	0.056	−6.806	0.682	0.610	0.761	<0.001
AMH (μg/L)							
AMH <1.1	1 (reference)						
1.1 ≤AMH<4	0.124	0.115	1.083	1.132	0.905	1.419	0.279
AMH ≥4	0.255	0.12	2.118	1.291	1.020	1.636	0.034
AFC(*n*)							
AFC <5	1 (reference)						
5 ≤AFC <15	0.121	0.171	0.71	1.129	0.810	1.583	0.478
AFC ≥15	0.139	0.174	0.801	1.149	0.820	1.621	0.423
Basal FSH level (IU/L)	0.002	0.013	0.186	1.002	0.977	1.028	0.852
Basal LH level (mIU/ml)	0.009	0.008	1.113	1.009	0.994	1.024	0.266
Basal P level (ng/ml)	0.07	0.045	1.544	1.072	0.986	1.177	0.123
E2 level on trigger day (pg/ml)	−0.006	0.005	−1.098	0.994	0.985	1.004	0.272
*P* level on trigger day (ng/ml)	0.055	0.028	1.984	1.057	1.001	1.116	0.047
Gn starting dose (IU)							
Other dosage	1 (reference)						
150 IU	0.163	0.056	2.92	1.177	1.055	1.312	0.004
225 IU	0.138	0.062	2.232	1.149	1.017	1.297	0.026
Recombinant FSH dose	0.001	0.001	0.908	1.001	0.999	1.002	0.364
Recombinant FSH duration	0	0	−0.269	1.000	1.000	1.000	0.788
LH total dose	0	0	−1.544	1.000	1.000	1.000	0.123
GnRH-A							
Ganirelix	1 (reference)						
Cetrorelix	−0.091	0.049	−1.846	0.913	0.830	1.006	0.065
GnRH-a dose on trigger day	−0.053	0.272	−0.195	0.948	0.557	1.615	0.845
Trigger day LH/Gn day LH ≥2	−0.087	0.079	−1.101	0.917	0.785	1.070	0.271
Number of embryos for ET							
1 embryo	1 (reference)						
2 embryo	0.411	0.045	9.222	1.508	1.382	1.646	<0.001
Male information							
Male age	−0.009	0.005	−1.877	0.991	0.981	1.000	0.061

BMI, body mass index; AMH, anti-Mullerian hormone; AFC, antral follicle counting.

Ovarian reserve markers exhibited divergent impacts: while AMH ≥4 μg/L independently improved success (aOR = 1.29, 95% CI 1.02–1.64, *p* = 0.034), AFC categories showed no significance (*p* > 0.05), suggesting AMH's superior predictive value for live birth in antagonist cycles (31). Gn starting doses of 150 IU (aOR = 1.18, 95% CI 1.06–1.31) and 225 IU (aOR = 1.15, 95% CI 1.02–1.30) demonstrated incremental benefits, likely reflecting optimized follicular recruitment in high responders without triggering premature luteinization—a key advantage of antagonist flexibility (10).

Trigger-day progesterone elevation (aOR = 1.06 per ng/ml, *p* = 0.047) and dual embryo transfer (aOR = 1.51, 95% CI 1.38–1.65, *p* < 0.001) were critical modifiable factors. Elevated progesterone may enhance endometrial receptivity (17), whereas dual transfers counterbalance antagonist-associated follicular asynchrony (24). Importantly, transferring two embryos significantly increased live birth odds by 51% compared to single embryo transfer (aOR = 1.51, *p* < 0.001), aligning with studies demonstrating improved cumulative pregnancy rates with dual transfers despite potential risks of multiple gestations (16, 17).

Ganirelix and Cetrorelix perform comparably in terms of live birth rates and embryo quality, yet their safety profiles diverge significantly. Cetrorelix excels in LH control and OHSS prevention, making it a reliable and safer choice for at-risk patients, while Ganirelix may suit specific cases requiring swift LH suppression. Clinical decisions should integrate individual patient characteristics, balancing efficacy and safety to achieve optimal reproductive outcomes. These insights underscore Cetrorelix's reliability in antagonist protocols, particularly for its favorable impact on endometrial morphology and reduced OHSS risk, aligning with the pursuit of safer, more effective ART strategies.

## Data Availability

The raw data supporting the conclusions of this article will be made available by the authors, without undue reservation.
